# Biodegradable and Ultra-High Expansion Ratio PPC-P Foams Achieved by Microcellular Foaming Using CO_2_ as Blowing Agent

**DOI:** 10.3390/nano14131120

**Published:** 2024-06-29

**Authors:** Change Wu, Tianwei Zhang, Jiaxin Liang, Jingyao Yin, Min Xiao, Dongmei Han, Sheng Huang, Shuanjin Wang, Yuezhong Meng

**Affiliations:** 1 The Key Laboratory of Low-Carbon Chemistry & Energy Conservation of Guangdong Province/State Key Laboratory of Optoelectronic Materials and Technologies, School of Materials Science and Engineering, Sun Yat-sen University, Guangzhou 510275, China; wuche@mail2.sysu.edu.cn (C.W.); zhangtw7@mail2.sysu.edu.cn (T.Z.); liangjx27@mail2.sysu.edu.cn (J.L.); yinjy3@mail2.sysu.edu.cn (J.Y.); stsxm@mail.sysu.edu.cn (M.X.); huangsh47@mail.sysu.edu.cn (S.H.); 2 School of Chemical Engineering and Technology, Sun Yat-sen University, Guangzhou 510275, China; handongm@mail.sysu.edu.cn; 3 Institute of Chemistry, Henan Academy of Sciences, Zhengzhou 450052, China; 4 Research Center of Green Catalysts, College of Chemistry, Zhengzhou University, Zhengzhou 450001, China

**Keywords:** poly(propylene carbonate-co-phthalate) (PPC-P), CO_2_ foaming, biodegradable foam, high expansion ratio, low-cost foam, packaging materials

## Abstract

Poly(propylene carbonate-co-phthalate) (PPC-P) is an amorphous copolymer of aliphatic polycarbonate and aromatic polyester; it possesses good biodegradability, superior mechanical performances, high thermal properties, and excellent affinity with CO_2_. Hence, we fabricate PPC-P foams in an autoclave by using subcritical CO_2_ as a physical blowing agent. Both saturation pressure and foaming temperature affect the foaming behaviors of PPC-P, including CO_2_ adsorption and desorption performance, foaming ratio, cell size, porosity, cell density, and nucleation density, which are investigated in this research. Moreover, the low-cost PPC-P/nano-CaCO_3_ and PPC-P/starch composites are prepared and foamed using the same procedure. The obtained PPC-P-based foams show ultra-high expansion ratio and refined microcellular structures simultaneously. Besides, nano-CaCO_3_ can effectively improve PPC-P’s rheological properties and foamability. In addition, the introduction of starch into PPC-P can lead to a large number of open cells. Beyond all doubt, this work can certainly provide both a kind of new biodegradable PPC-P-based foam materials and an economic methodology to make biodegradable plastic foams. These foams are potentially applicable in the packaging, transportation, and food industry.

## 1. Introduction

In recent years, polymer foams have become much more irreplaceable in modern society due to their known merits, such as unique porous structure and excellent comprehensive performance, including lightweight, high impact strength, good heat, and sound insulation. However, traditional foams such as polypropylene (PP) foams, polystyrene (PS) foams, etc., are from non-renewable petroleum resources, and they can hardly degrade in the natural environment. Biodegradable polymer foams from renewable resources, thus, have emerged as the “green environmental” requires; for example, biodegradable foams like poly(lactic acid) (PLA) [1] and poly(propylene carbonate) (PPC) [2] have been tried to use as substitutes of the petroleum-based polymer foams. They have well-known merits, such as relatively comparable impact strength, superior flexibility, and excellent biodegradability and renewability. However, their comparatively low melt strength and heat resistance have constrained their industrial use, which can be surmounted through modifications such as hybridization with high-stiffness polymers by introducing crystallization, adding fillers, or through graft branches [3,4,5].

Poly(propylene carbonate) (PPC) is obtained through copolymerization of carbon dioxide (CO_2_) and propylene oxide (PO) [6]. Due to that its raw materials mainly are carbon dioxide and the obtained polymers are fully biodegradable, it is becoming increasingly popular and receiving attention around the world. Besides, PPC has good oxygen insulation performance, excellent transparency and processing performance, and high flexibility with an elongation at break up to 1200%. It is widely considered to be used in the foam field [7]. PPC/CaCO_3_ foams with well-developed and uniform cellular structures were obtained through the chemical foaming method in order to improve PPC foams’ compressive strength by introducing CaCO_3_ fillers [2]. It has also been suggested that PPC has higher solubility in CO_2_ compared with other biodegradable materials [8]; at the same saturation condition, PPC showed a 23.1 wt% CO_2_ absorption capability, while PBAT only absorbed 5.0 wt% CO_2_. Therefore, better resilient PPC/PBAT hybrid foams can also be fabricated by using CO_2_ as the physical blowing agent. However, due to the low glass transition temperature (Tg ~37 °C) and poor mechanical properties of PPC (Tensile strength ~20 MPa), its application is greatly limited. Thus, it is always challenging to prepare biodegradable PPC foams with a high foaming ratio foams and fined cellular structure, which often requires polymers to possess advanced melt strength, superior stiffness, and good heat resistance. The main obstacles of PPC are insufficient to compensate for the shortcomings of fast gas diffusion during the foaming process, resulting in cell collapse and inferior cellular structure. There are many strategies to improve PPC’s melt strength, thermal properties, and foaming abilities. Firstly, fillers such as calcium carbonate (CaCO_3_), carbon black, graphite oxide, nano-cellulose, starch, and carbon nanotubes (CNTs) are added [9,10]. The addition of nano-CaCO_3_ into the PPC matrix improved the thermal properties and foaming abilities of PPC [11]; notably, the finest cell structure with a narrow cell diameter distribution was obtained at a 3 wt% nano-CaCO_3_ content. On the other hand, blending modification with other degradable polymers such as polylactic acid (PLA) [1], polybutylene succinate (PBS) [12], and PBAT were investigated extensively [13,14,15]. The compressive modulus of prepared PPC/PBS/PTFE (70/30/3) foams was 30-fold higher than that of neat PPC foams [16]. Nevertheless, it still had inferior cell quality.

Additionally, incorporating aromatic polyester units into the PPC backbone to tune comprehensive properties has attracted much more interest from researchers [17]. PPC-P (PO/PA/CO_2_ terpolymers) is an amorphous aliphatic polycarbonate-co-aromatic polyester synthesized from the alternative copolymerization of propylene oxide (PO), with CO_2_ and phthalic anhydride (PA). The chemical structures of PPC and PPC-P are shown in Scheme S1. After introducing aromatic PA units, PPC-P exhibits comparatively higher *T_g_* (>45 °C) and tensile strength (>40 MPa) than PPC, and their values can be tailored by varying the feed ratio of PA and PO [6,18], which are comparable to crystalline polymer PLA. Moreover, PPC-P exhibits satisfactory biodegradability when compared with commercial PBAT and PPC. Based on the above properties, PPC-P is expected to be used to produce fully biodegradable foams with a high foaming ratio and fine cellular structure.

To our knowledge, no research on the foaming behaviors of PPC-P has been disclosed because PPC-P has been reported and commercialized by our group very recently. In this work, we first prepare PPC-P foams with different expansion ratios by using the autoclave foaming method with sub-critical CO_2_ as the blowing agent. Followingly, we investigate how the saturation pressure and foaming temperature affect the foaming behaviors of PPC-P, including CO_2_ adsorption and desorption performance, the foaming ratio, cell density, cell size, and porosity. Furthermore, the influences of nano-CaCO_3_ and starch fillers are studied on the rheological properties of PPC-P polymer and the cellular structures of PPC-P foams. The intent of this work is to provide new insights into how to prepare biodegradable PPC-P foams and low-cost PPC-P-based composite foams with high foaming ratios and fine cellular, together with their practical applications.

## 2. Materials and Methods

### 2.1. Materials 

PPC-P (*M*_n_ = 124 kg/mol, PDI = 2.2) was supplied by Shandong Lianxin Environmental Protection Technology Co., Ltd. (Maoming, China). The melt flow rate and glass transition temperature are respectively 20.1 g/10 min (at 190 °C/2.16 kg load) and ~46 °C. Nano calcium carbonate(nano-CaCO_3_) was purchased from Macklin company (Shanghai, China) and has an average size of 50 nm. The starch was provided by Guangzhou Jinling Biotechnology Co., Ltd. (Guangzhou, China). All of them were dried at 80 °C under a vacuum for 24 h before use. Commercial-purity-grade high-pressure CO_2_ (purity = 99.9%, Guangzhou, China) was used as a physical blowing agent.

### 2.2. Fabrications of Polymeric Foams [19]

The foaming process can be divided into the following steps [19]. Firstly, the PPC-P specimens are pressed into sheets with a thickness of approximately 1 mm on a hot plate at 150 °C. And the specimens with a size of 1.0 cm × 1.0 cm are cut for gas solubility measurement. Next, PPC-P sheets are sealed into an autoclave and are flushed with low-pressure CO_2_ for about 2 min and then saturated with CO_2_ at 2.5–4.0 MPa. After the saturation treatment for 24 h, the autoclave is cooled in an ice water bath until the temperature is reduced to around 0 °C to avoid the pre-foaming phenomenon due to the sharp plasticization effect of CO_2_ on PPC-P. 

After the cooling process, the CO_2_-saturated sheets are removed from the autoclave after a quick depressurization and then are rapidly put into a boiling-water bath for 60 s to complete the foaming process. Finally, the foamed sheets are transferred into the ice-water bath again to stabilize the porous structures. The obtained foam is named PPC-P-*x* foam (*x* represents the saturation pressure of CO_2_ before foaming). After experimental optimization, the foaming temperature is set as 50 °C or 60 °C.

**Scheme 1:**
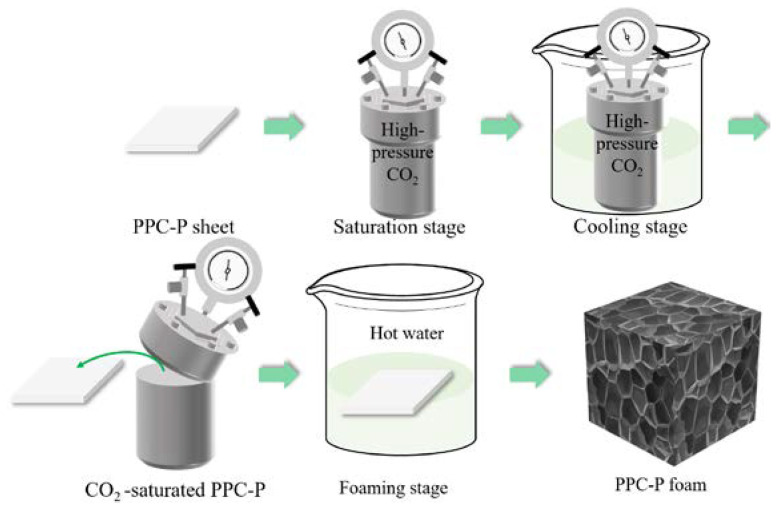
The fabrication process of PPC-P-based foams.

Therefore, Scheme 1 depicts the autoclave foaming process with sub-critical CO_2_ as a blowing agent.

### 2.3. Microstructure and Morphology Characterization

A TESCAN VEGA3 SEM is used to analyze the microscopic cellular structure of the fabricated foamed samples. Before the SEM observation, all the foams are first freeze-fractured after being immersed in liquid nitrogen for 10 min, and then the fracture surfaces are coated with a layer of Ag. A 5 kV accelerating voltage and the secondary electron image mode are used to take all SEM images.

The water displacement method [20] is utilized to measure the density of the solid (ρ) and foam samples (ρ_f_), and the expansion volume ratio R_e_ could be calculated by Equation (1):

R_e_ = ρ/ρ_f_, (1)

where ρ and ρ_f_ are the density of the solid and foams, respectively. At least three foams are selected to measure, and the average values are analyzed for the foams obtained under the same conditions. The following tests also obeyed such principle.

The foams’ porosity (ε) is calculated based on the following Equation (2):

Porosity (ε) = (1 − 1/R_e_) × 100%, (2)

where R_e_ is the foams’ expansion volume ratio.

Both the cell diameter and density are determined from the SEM (SEM, SU8010, Hitachi, Tokyo, Japan) micrographs with nano-measure 1.2 software. The cell density (*N*_0_; cells/cm^3^) and nucleation density (*N_d_*; nuclei/cm^3^) are counted from Equations (3) and (4):

Cell density (*N*_0_) = ε ÷ [4/3 π(d/2)^3^] = 6ε/(πd^3^), (3)

nucleation density (*N_d_*) = *N*_0_/ρ_f_, (4)

where *d* is the average cell diameter (μm) of foams.

### 2.4. CO_2_ Adsorption and Desorption Measurement

The CO_2_ adsorption and desorption behaviors of PPC-P sheets are measured by a widely utilized gravimetric method. The PPC-P sheets are placed in a high-pressure autoclave with 50 mL volume at room temperature and 40 °C. The autoclave is flushed with compressed CO_2_ for 2 min and then saturated at different pressures of 2.5–4.0 MPa. All the PPC-P sheets are sealed into the autoclave for 24 h to ensure an equilibrium adsorption of CO_2_. After the CO_2_ saturation process, the pressure is released, and the specimen is transferred rapidly from the autoclave to a digital balance within 1 min to record the mass loss as a function of time. The mass adsorption of CO_2_ in the high-pressure autoclave is calculated by linear extrapolation of the initial stage of the desorption curve of CO_2_. The samples’ initial masses are recorded as *M*_0_ before the samples are placed into the autoclave. Next, the samples are quickly transferred to the analytical balance after depressurization, and the samples’ mass is documented as *M*_t_ as the time t varies. Then, the CO_2_ adsorption mass in the polymer can be expressed as (*M*_t_ − *M*_0_), and the CO_2_ adsorption capability in the sample can be expressed as (*M*_t_ − *M*_0_)/*M*_0_, simply named as *m*_t_.

The desorption diffusion coefficient (*D*) of PPC-P polymers is calculated by the following Equation (5) [21]:

*m*_t_/*m*_∞_ = 1 − 4/h (*D*t/π)^0.5^, (5)

where *m*_∞_ refers to the solubility obtained by linear extrapolation based on the desorption curve, h is the polymer sheet’s thickness, t is the desorption time, and *D* represents the gas diffusion coefficient.

We provide other fabrications and characterization techniques of various blends and foams in the (Supplementary Information File). 

## 3. Results and Discussions

### 3.1. Solubility and Diffusivity of CO_2_ in PPC-P

**Figure 1:**
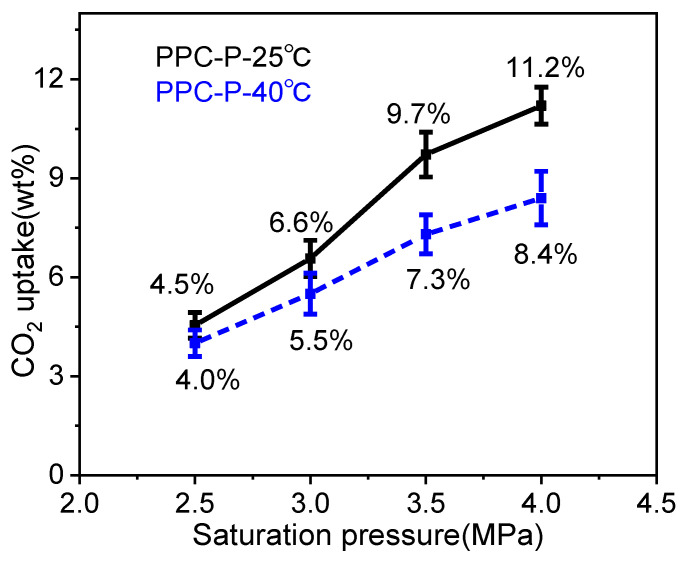
CO_2_ uptakes of PPC-P sheets with different saturation temperatures and pressures.

The CO_2_ absorption capability for PPC-P is presented in Figure 1. The absorption behaviors under different saturation pressures and various saturation temperatures were investigated since the gas solubility in the polymer and blowing agent system directly determines cell nucleation and growth [22]. The low gas solubility and inferior plasticization effect of physical blowing agents have been proven as the main obstacles to preparing low-density and high-performance polymer foams. Therefore, the co-blowing agent with compressed CO_2_ together is always used to enhance CO_2_ solubility in polymers [23], while the pure PPC polymer, due to over 40 wt% of CO_2_ content, exhibits significantly higher CO_2_ solubility than most other polymers. As an analog of PPC, PPC-P theoretically is also expected to possess superior CO_2_ absorption capability, as illustrated in Scheme S2. It is testified that PPC-P can absorb the highest 11.2 wt% CO_2_ under 4.0 MPa and at 25 °C room temperature. When the saturation pressure decreases, it is found that the uptake of CO_2_ decreases rapidly. Surprisingly, even under 3.0 MPa saturation pressure, 6.6 wt% CO_2_ absorption can also be achieved in the PPC-P, which can trigger sufficient cell nucleation and further prepare foams with a high expansion ratio, in which the expansion ratios can reach up to 19 times. Saturation temperature also affects the solubility of CO_2_ in PPC-P. As the saturation temperature increases from 25 °C to 40 °C at 3.5 MPa, the CO_2_ absorption decreases from 9.7 wt% to 7.3 wt%. Moreover, it can be seen that the relationship between the pressure and the CO_2_ solubility is approximately linear, which agrees with Henry’s law [24]. The Henry constant, which is gained from the curve’s slope, decreases with increasing the foaming temperature, as presented in Table 1. To be specific, the Henry constants at 25 and 40 °C saturation temperature are respectively 4.6 and 3.1 (100/MPa). In general, the higher Henry constant means higher CO_2_ absorption capability. Therefore, elevating the saturation temperature weakens the CO_2_ absorption capability [25]. That is probably explained by the higher temperature affecting the movements of molecular chains, thereby leading to the decline of CO_2_ solubility. Besides, the Henry constant of CO_2_ in PPC-P polymer is significantly higher than that of PBAT polymer, further verifying the excellent affinity between CO_2_ and PPC-P. In conclusion, due to its unique molecular structure, PPC-P exhibits outstanding CO_2_ adsorption capacity under low saturation pressure, which provides the possibility to produce low foam density and a high foaming ratio of the foams.

**Figure 2:**
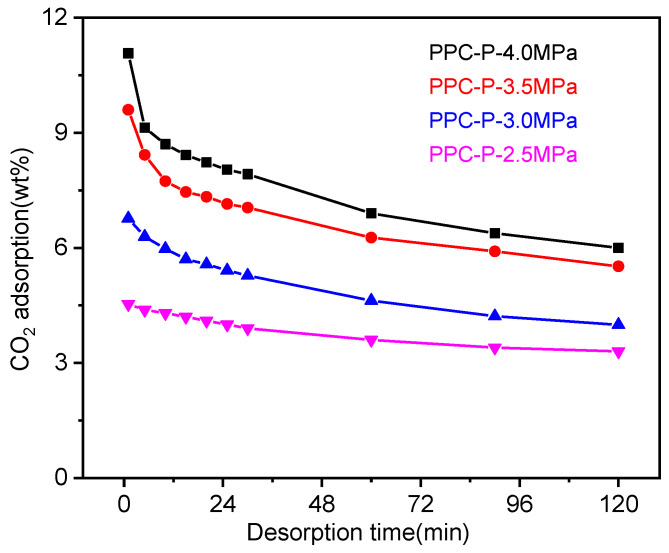
CO_2_ desorption profiles of PPC-P sheets with different saturation pressures at 25 °C.

Rapid gas escape is typically characteristic of a CO_2_-blowing agent for polymer foaming. Hence, we also studied the CO^2^ diffusion behaviors in PPC-P polymer under varied saturation pressure, as shown in Figure 2. Based on Equation (5), the diffusion coefficient *D* can be easily obtained from the slope of this graph. Obviously, it is evident that the CO_2_ diffusion coefficient in PPC-P under 4.0 MPa saturation pressure is the highest one, as demonstrated in Table 2. The CO_2_ diffusion coefficient decreases accordingly when the saturation pressure increases. For instance, the CO_2_ diffusivity at 4 MPa is 8.06 × 10^−9^ m^2^⋅s^−1^, which is 2 orders of magnitude higher than that of diffusivity at 2.5 MPa. This is because the plasticization of CO_2_ blowing agent is more obvious for the saturation of 4.0 MPa; thereby, the *T*_g_ of PPC-P is much lower than the room temperature. Hence, the PPC-P/CO_2_ solution at 4.0 MPa is in an extremely unstable state after removing it from the autoclave. It can also be seen that the CO_2_ uptake decreases quickly within 10 min, and the residual CO_2_ uptake still remains 8.0 wt% after 30 min, which means that there is still enough foaming agent to gain high cell density foams. Finally, 46 wt% of the CO_2_ mass escaped, and the residual CO_2_ was about 6.0 wt% in 120 min, as shown in Figure 2. As the saturation pressure decreases, the CO_2_ diffusion coefficient decreases accordingly. Especially at 2.5 MPa saturation pressure, only 27 wt% CO_2_ escapes in 120 min. In other words, the rapid CO_2_ diffusion can be observed when the CO_2_ uptake is high in PPC-P.

### 3.2. Foaming Behavior of PPC-P Using Sub-Critical CO_2_ as Foaming Agent

According to the cell formation mechanism, physical foaming technology can be divided into pressure quench foaming and temperature rising foaming by using CO_2_ as a blowing agent [15,26]. The main difference is the method of bubble nucleation and cell growth. For temperature-rising foaming, the bubble nucleation and cell growth are triggered by the rapid drop in the CO_2_ solubility in the polymer due to the increased temperature (above the *T*_g_). By varying the foaming temperature, the viscosity of the polymer can be changed, resulting in different foaming behaviors. While for pressure quench foaming, a sudden pressure drop is the induced parameter, by varying the depressurization rate, various foaming behaviors can be obtained [27]. Besides, pressure quench foaming is common in polymer systems, especially polymers with high crystallinity. Both foaming technologies are befitted to prepare foams of amorphous polymers. Because of the low cost and convenience of the operation process for the temperature-rising foaming rather than the pressure quench foaming, we utilize the temperature-rising foaming process to prepare PPC-P-based foams.

#### Influences of Pressure and Temperature on Foam Morphology

**Figure 3:**
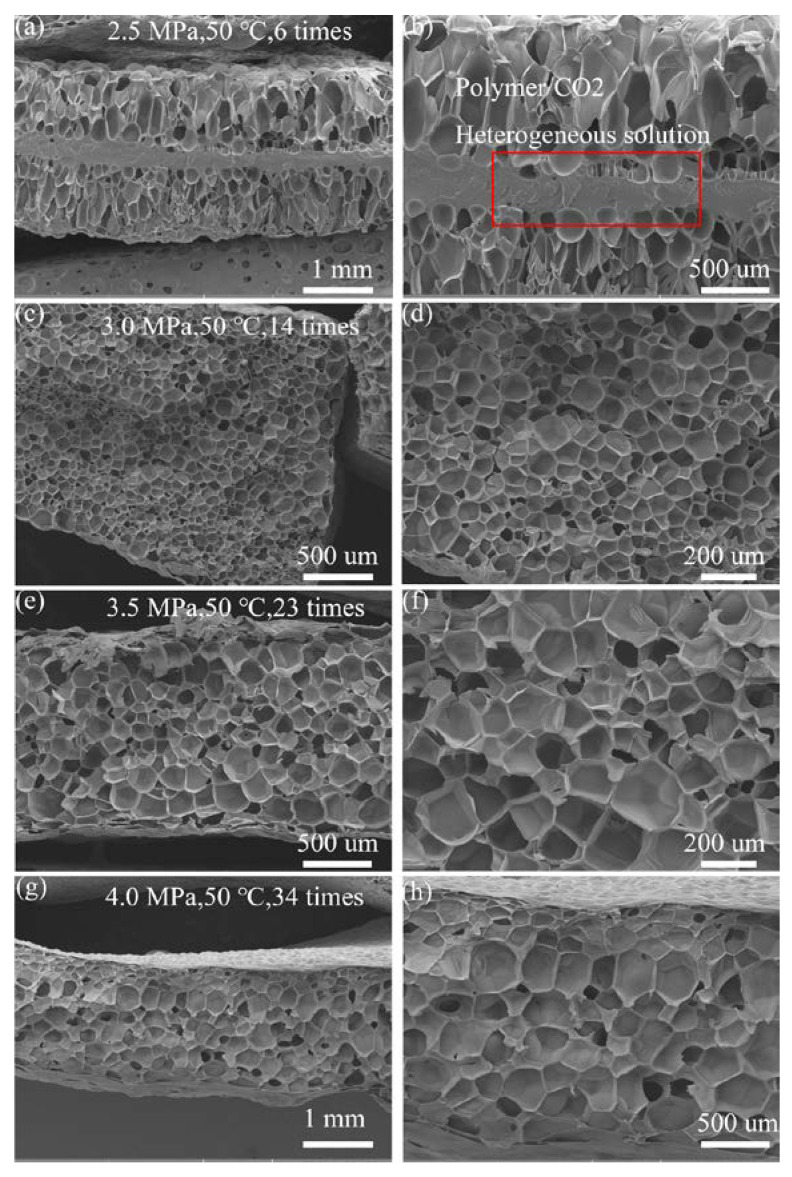
SEM images of cross-sections of PPC-P foams obtained at 50 °C with different saturation pressures: (**a**,**b**) 2.5 MPa, (**c**,**d**) 3.0 MPa, (**e**,**f**) 3.5 MPa, and (**g**,**h**) 4.0 MPa. Enlarged images in (**b**,**d**,**f**,**h**) show the details of the cell structure, i.e., more precise cell diameters can be calculated.

**Figure 4:**
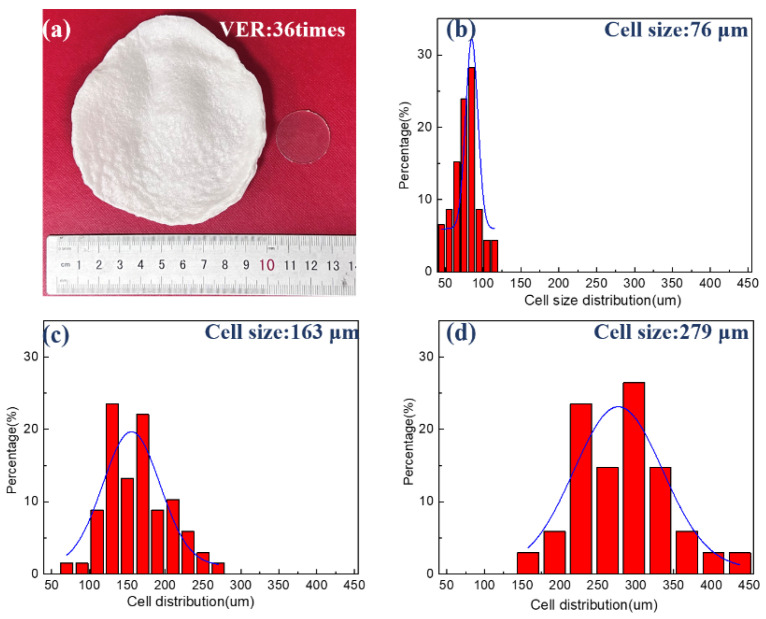
Cell size distributions of PPC-P foams obtained at 50 °C with different saturation pressures: (**a**) optical photograph of PPC-P foam with 36 VER, (**b**) 3.0 MPa, (**c**) 3.5 MPa, and (**d**) 4.0 MPa.

The saturation pressure greatly influences the uptake of CO_2_ in the polymer prior to foaming. Generally, the poor CO_2_ solubility results in the low uptake of foaming agents and tends to form heterogeneous phases, which is not conducive to preparing foams with uniform and fine cell structures [28]. According to homogeneous nucleation theory, higher uptake of a foaming agent implies greater super-saturation degree and lower nucleation barrier; hence, higher cell density can be achieved in the foaming product [29]. Because of the excellent affinity between CO_2_ and PPC-P, the PPC-P/CO_2_ homogeneous solution can be obtained under relatively low pressures. Therefore, the PPC-P sheets are saturated by CO_2_ at different pressures of 2.5–4.0 MPa and then foamed at 50 °C for 60 s. Figure 3 shows the cell morphologies of the PPC-P foams with different volume expansion ratios (VER), and the cell size distributions are presented in Figure 4. Furthermore, the corresponding average cell size, porosity, cell density, and nucleation density are summarized in Figure S3 and Table S1. At first, the un-foamed fractions are found in the cross-section of PPC-P foam because of the insufficient CO_2_ uptake at 2.5 MPa saturation pressure for 24 h, hereby leading to uneven and un-fully foaming behavior as shown in Figure 3a,b. When the saturation pressure increases, much more uniform cell morphology is observed. Besides, due to the increase of CO_2_ uptake from 2.5 MPa to 4.0 MPa of saturation pressure, the VER values of PPC-P foam significantly increase from 6 times to 34 times at 50 °C. Meanwhile, the cell size and porosity increase obviously with increasing the CO_2_ uptake, i.e., from 76.1 to 278.5 μm and from 92.9 to 97.1%, respectively. According to the cell growth theory, that is probably because the earlier produced small pores tend to collapse and merge when the much more physically trapped CO_2_ diffuses during the foaming procedure [30].

Figure 4a depicts the optical photograph of PPC-P foam with 36 times VER, which possesses a pretty appearance and cell quality. As shown in Figure 4b–d, the cell size distributions of all foams nearly obey the Gaussian distribution, and a narrower distribution width can be found for the PPC-P-3.0 foam (Figure 4b). With the increase of the saturation pressure, the cell size distribution becomes much broader. It is worth mentioning that the high cell densities of those foams are obtained even with such high VER. For the PPC-P-4.0 foam with 34 times VER, its cell density is 0.9 × 10^7^ cell/cm^3^, while for the one with 23 times VER, its cell density increases to 4.2 × 10^7^ cell/cm^3^. With further decreasing CO_2_ saturation pressure, the cell density increases up to 4.0 × 10^8^ cells/cm^3^ for PPC-P-3.0 foam. More importantly, the nucleation density also increases to 4.5 × 10^9^ nuclei/cm^3^. Compared with the PPC foams described in reference [31], great progress has been made in the cell quality of PPC-P foams. In conclusion, a fully “green,” high cell density, and VER foams can be readily fabricated by using sub-critical CO_2_ as a physical blowing agent.

**Figure 5:**
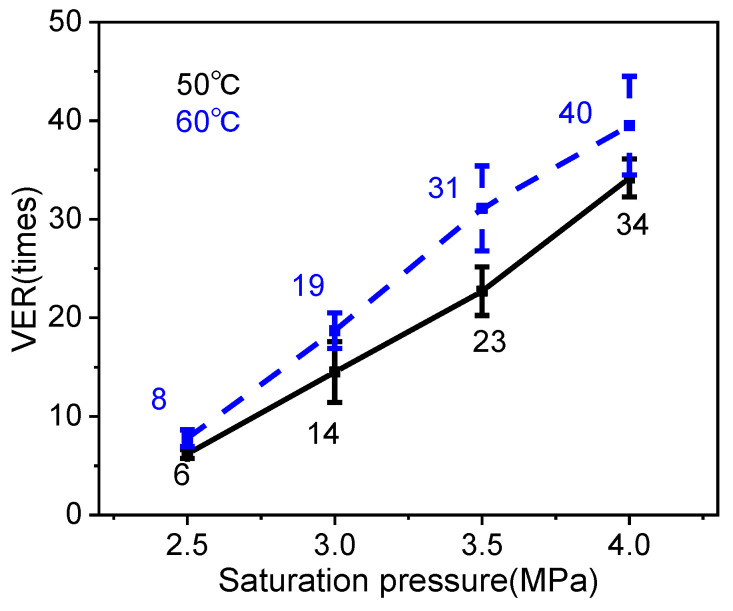
VER profiles versus saturation pressure of PPC-P obtained at the foaming temperature of 50 and 60 °C.

Foaming temperature critically decides the foaming window of a polymer. Due to the glass transition temperature (*T*_g_) of PPC-P being slightly higher than room temperature and because of the plasticization of the CO_2_ blowing agent, the *T*_g_ value will inevitably decrease after CO_2_ saturation [32]. Thus, the foaming window is relatively narrow. The results can also be further verified. The PPC-P sheets cannot be fully foamed at a lower temperature of 40 °C, and the samples have serious shrinkage at higher temperatures of 70 °C during the foaming process. The satisfactory foam samples can be obtained only at the foaming temperature of 50 °C or 60 °C, and their VER profiles versus saturation pressures are depicted in Figure 5. The PPC-P foams show an apparent increase of the VER with increasing temperature. For instance, the VER of PPC-P-3.5 increases evidently from 23 to 31 times by elevating the foaming temperature from 50 to 60 °C. This is because the faster CO_2_ gas diffusion rate at higher foaming temperatures facilitates cell growth. It should be noted that the average VER can reach up to 40 times with the 4.0 MPa saturation pressure and at the 60 °C foaming temperature. However, the error bars of VERs of the foams obtained at 60 °C and high saturation pressure conditions are greater than those of the foams obtained at 50 °C. This can be explained by the high-temperature incline, which easily overcomes the potential energy of cell nucleation and puts PPC-P/CO_2_ samples in an unstable state when the uptake of the blowing agent is high enough. 

Figure S1 displays the cell morphologies, and Figure S2 gives cell size distributions of different PPC-P foams obtained at 60 °C. It can be observed that PPC-P-3.0 foam with VER of 18 times at a foaming temperature of 60 °C has a uniform cell morphology with negligible open cell structure. However, compared with the foams obtained at 50 °C, with increasing CO_2_ saturation pressure, much more open cell structure and larger cell size are observed due to abundant cells ruptured and reunited, which is attributed to the fast CO_2_ gas diffusion at higher foaming temperature. Shrunk cells even appear at a foaming temperature of 60 °C for the PPC-P-4.0 sample because the cell strength of PPC-P cannot suffer such a large cell size. The approximate Gaussian distribution of cell sizes at 60 °C can still be observed in Figure S2. With increasing average cell size, the cell size distribution becomes even broader. Compared with the foams obtained at 50 °C, both the cell sizes and porosities of the foams obtained at 60 °C increase, while the cell densities and nucleation densities decrease. For example, the largest average cell size (351.3 μm) and smallest cell density (4.3 × 10^6^ cells/cm^3^), as well as the smallest nucleation density (1.4 × 10^8^ nuclei/cm^3^), are achieved for PPC-P-4.0 foam at the foaming temperature of 60 °C according to Figure S3 and Table S1. It is suggested that with high pre-saturation pressure, the foaming temperature significantly affects the cell size, cell density, nucleation density, and morphology of the PPC-P foams. In fact, the accelerated movement of molecular chains at higher temperatures weakens the resistance of the polymer matrix to cell growth. The foam with a higher VER than 30 times is usually defined as ultralow-density foam [33]. In our study, ultralow-density PPC-P foams with more than 40 times VER can be fabricated delightfully.

### 3.3. Rheological Behavior and Mechanical Performance of PPC-P and PPC-P Composites

**Figure 6:**
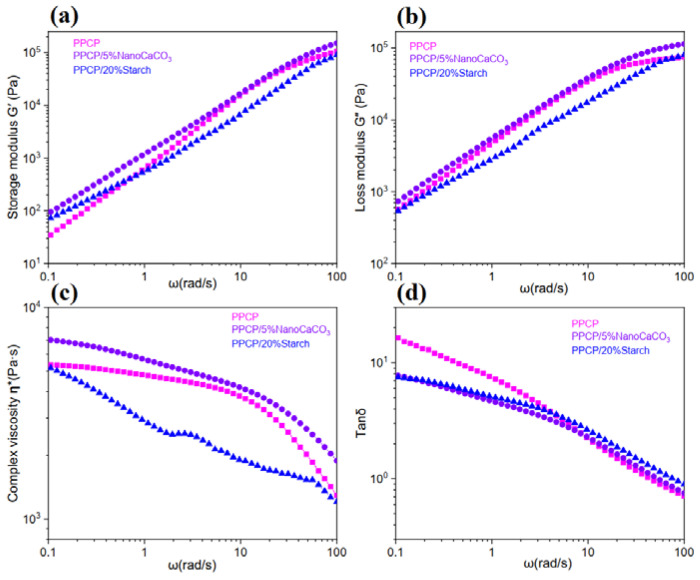
(**a**) Storage modulus (G′), (**b**) loss modulus (G″), (**c**) complex viscosity (η*), and (**d**) Tan*δ* of the neat PPC-P, PPC-P/5%nanoCaCO_3_ and PPC-P/20%starch composites as a function of ω at 150 °C.

The rheological behavior, especially at low frequencies, is very susceptible to the variation of the microstructure of the filled polymer composites; thereby, the rheological properties of the neat PPC-P and modified PPC-P are investigated in detail. Figure 6a–c shows the variations in dynamic storage modulus G′, loss modulus G″, and complex viscosity η* as a function of frequency ω. At low frequencies, the PPC-P/5%nanoCaCO_3_ composite exhibits the highest G′, G″, and η*. When compared with pristine PPC-P, the G′ and η* of PPC-P/5%nanoCaCO_3_ composite increase by about 280% and 130% respectively at ~0.1 rad/s. It is generally believed that the high G′ can advantageously support cell growth and obtain polymer foams with high VERs. Unexpectedly, although the storage modulus of PPC-P/20%starch composite is higher than that of the pristine PPC-P at a frequency of 0.1 rad/s, the complex viscosity is lower instead, which might be caused by the high content of amylose in starch [34]. The damping factor Tan *δ* is equal to G″/G′. When the value of Tan δ increases, the viscoelasticity of polymers improves, and the foaming ability of polymers is expected to enhance [35]. As Figure 6d shows, all PPC-P composites exhibit improved viscoelasticity compared with pristine PPC-P at low frequencies. Besides, the tensile properties of neat PPC-P and modified PPC-P are also assessed, as shown in Figure S4. Consistent with the results of storage modulus, the PPC-P/5%nanoCaCO_3_ composite exhibits the highest tensile strength due to the enhancement of nano-fillers.

### 3.4. Foaming Behaviors of PPC-P Composites

The incorporation of inorganic or biomass materials, such as nanoCaCO_3_ and starch, by simple blending method, can obviously reduce the cost of biodegradable polymers. The presence of those fillers can also improve the melt strength and affect the foaming behaviors of the polymer matrix. Moreover, the fillers can act as nucleating agents to affect the distribution of the gas phase and then control the foams’ morphology. Hence, fully biodegradable and low-cost PPC-P composite foams with adjustable microcellular structures are fabricated using sub-critical CO_2_ as a blowing agent by introducing nanoCaCO_3_ and starch as fillers, respectively.

#### 3.4.1. Foaming Behavior of PPC-P/5%nanoCaCO_3_ Composite

**Figure 7:**
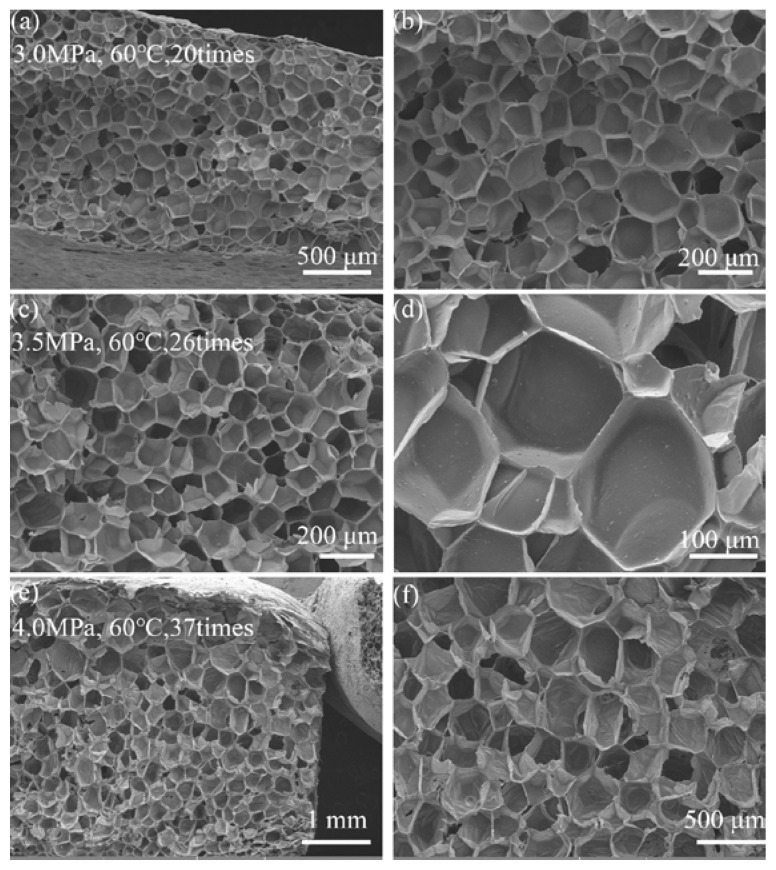
SEM images of cross sections of PPC-P/5%nanoCaCO_3_ foams at 60 °C foaming temperature and different saturation pressures: (**a**,**b**) 3.0 MPa, (**c**,**d**) 3.5 MPa, and (**e**,**f**) 4.0 MPa. Enlarged images in (**b**,**d**,**f**) show the details of the cell structure.

The influences of saturation pressure on the foaming behavior of PPC-P/5%nanoCaCO_3_ composite are also investigated, in which the composite sheets are foamed at 60 °C after being saturated with CO_2_ under pressure of 3.0–4.0 MPa. Figure 7 and Figure S6 display the SEM images and cell distributions of PPC-P/5%nanoCaCO_3_ foams under different saturation pressures. The corresponding average cell sizes, porosities, cell densities, and nucleation densities are summarized in Figure S5 and Table S1. Firstly, the CO_2_ uptakes of PPC-P/5%nanoCaCO_3_ sheets are slightly lower than those of the pristine PPC-P sheets under different saturation pressures (for example, the change is from 9.7% to 8.2% for 3.5 MPa saturation pressure, Table S1). With the increase of saturation pressure from 3.0 MPa to 4.0 MPa, the CO_2_ uptake of the PPC-P/5%nanoCaCO_3_ sheet increases from 6.0 to 9.8 wt%. As a result, after foaming at 60 °C, the VER increases from 20 to 37 times, and the cell size increases from 124.9 μm to 330.4 μm, correspondingly for PPC-P/5%nanoCaCO_3_ foam. Pointedly, the PPC-P/5%nanoCaCO_3_-3.5 foam with VER of 26 times has the smallest cell size and the largest cell density as well as the largest nucleation density, 110.0 μm, 1.4 × 10^8^ cells/cm^3^ and 2.9 × 10^9^ nuclei/cm^3^ respectively. While the pristine PPC-P foam with the close VER of 23 times shows bigger cell size and lower cell density as well as lower nucleation density of about 163.4 μm, 4.2 × 10^7^ cells/cm^3^, and 7.7 × 10^8^ nuclei/cm^3^, respectively, indicating that the presence of nanoCaCO_3_ filler improves the foaming behavior of PPC-P. That is because nanoCaCO_3_ can act as heterogeneous nucleation sites where cell nucleation happens at a low level of free energy during foaming.

On the other hand, the PPC-P/5%nanoCaCO_3_ foams show even narrow cell diameter distribution, as shown in Figure S6. It also can be confirmed by comparing the cell morphologies of the PPC-P/5%nanoCaCO_3_ foams with those of the pristine PPC-P foams. The former exhibits a much more uniform cell structure. In addition, PPC-P/5%nanoCaCO_3_ foams show fewer open cell structures under higher pressure than the pristine PPC-P foams, as shown in Figure S1. Presumably, it results from the enhanced modulus of PPC-P/5%nanoCaCO_3_ compared to that of the pristine PPC-P. Combined with the compressive strength of foams, as presented in Table S2, the added nanoCaCO_3_ can significantly improve the compressive strength of PPC-P foams. Oppositely uniform dispersion of nanoCaCO_3_ particles also can be observed in Figure 7d. Thus, the added nanoCaCO_3_ can effectively ameliorate the foams’ cellular structure, decrease the cell diameter, and increase cell density.

#### 3.4.2. Foaming Behavior of PPC-P/20%Starch Composite

**Figure 8:**
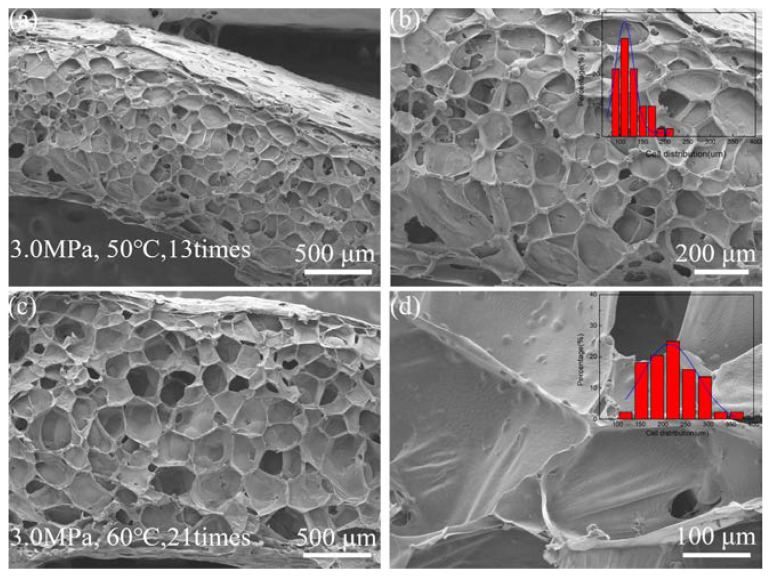
SEM images of cross sections of PPC-P/20%starch foams under 3.0 MPa of CO_2_ saturation pressure at different foaming temperatures: (**a**,**b**) 50 °C and (**c**,**d**) 60 °C. Enlarged images in (**b**,**d**) show details of cell structure. The inserts in (**b**,**d**) show the cell size distributions of PPC-P/20%starch foams.

It is well known that starch, as a natural, fully biodegradable polysaccharide, has been broadly studied due to its low cost [36,37]. However, its poor melt processing properties limit starch’s extensive application. Therefore, plasticizers are always utilized to modify starch. In our present work, the PPC-P/20%starch composite is fabricated successfully due to the excellent processing performance of PPC-P. Following this, the influence of starch content on the foaming behavior of PPC-P is studied. Figure 8 displays the SEM images and cell distributions of PPC-P/20%starch foams at different foaming temperatures and under 3.0 MPa of CO_2_ saturation pressure. The average cell sizes, porosities, cell densities, and nucleation densities are summarized in Table S1. With the increase of the foaming temperature from 50 °C to 60 °C, the VER and cell size of PPC-P/20%starch foam increases from 13 to 21 times and from 124.6 μm to 217.6 μm respectively. Also, with the increase of VER, the cell distribution becomes much wider. We can also find that the cell size of PPC-P/20%starch foams is larger than those of pristine PPC-P foams with the close VER; for instance, the cell size of PPC-P/20%starch foam is about 124.6 μm with 13 times of VER, while the PPC-P foam has only about 76.1 μm of cell size with 14 times of VER.

Figure 8 shows that abundant open cells are found in the cell morphologies of PPC-P/20%stach foams, while the closed cells are the majority structure for both PPC-P and PPC-P/5%nanoCaCO_3_ foams. Expectedly, PPC-P/20%starch foams with enough open cells probably can be used for petroleum oil spill clean-up. Moreover, the produced open cell structure presumably ascribed to the inferior complex viscosity of PPC-P/20%starch composite. Therefore, a large number of blowing gases coalesced and collapsed, resulting in the appearance of large cell size and open cell structure. Uniform and large starch particles can be obviously found in the foams, as shown in Figure 8b,d. According to heterogeneous nucleation theory, the aggregated and uneven micro-particles, which have a low surface-to-volume ratio, then reduce the number of heterogeneous nucleation sites during foaming. This is embodied in the decreased nucleation density compared with the pristine foams of the close VERs. As a result, the PPC-P/20%starch foams have large cell sizes and low cell density.

## 4. Conclusions

For the first time, fully biodegradable PPC-P foams with different VERs and refined cellular structures can be fabricated via the autoclave foaming method by using sub-critical CO_2_ as a physical blowing agent. It is demonstrated that the increased saturation pressure can availably increase the CO_2_ uptake to 11.2 wt% under 4.0 MPa. Moreover, the VER of PPC-P foam can reach up to 40 times at the 60 °C foaming temperature and under the 4.0 MPa saturation pressure. The optimal foaming conditions are under 3.5 MPa and at 60 °C. The foaming ratio, average cell size, cell density, and nucleation density, respectively, are 31 times 260.3 μm,1.1 × 10^7^ cells/cm^3^, and 2.6 × 10^8^ nuclei/cm^3^ in turns. In addition, by adding nanoCaCO_3_ filler, the storage modulus and complex viscosity of PPC-P increase by 280% and 130%, respectively, at ~0.1 rad/s, which are beneficial to improve the foaming properties of PPC-P. It can be verified that the PPC-P/5%nanoCaCO_3_ foam with 26 times VER has a smaller cell size and larger cell density as well as larger nucleation density than those of the pristine PPC-P foam with close to 23 times VER. Besides, the cell structure of PPC-P-based foams can be transformed from closed cell to enriched open cell by introducing 20%starch. In all, this work successfully provides a kind of new, fully biodegradable, and low-cost PPC-P-based foams that can be found in a well-broad application in food and packaging areas in the future.

## Figures and Tables

**Table 1:** Henry constant K of CO_2_ in PPC-P polymer under various saturation temperatures.

Saturation Temperature (°C)	K × 10^2^ (1/MPa)
25	4.6
40	3.1

**Table 2:** Diffusion coefficient of PPC-P polymer under various saturation pressures.

Saturation Pressure (MPa)	Diffusion Coefficient (m^2^ × s^−1^)
2.5	5.11 × 10^−11^
3.0	4.80 × 10^−10^
3.5	2.83 × 10^−9^
4.0	8.06 × 10^−9^

## Data Availability

Data are contained within the article and Supplementary Materials.

## Supplementary Materials

The following supporting information can be downloaded at: https://www.mdpi.com/article/10.3390/nano14131120/s1, Scheme S1: Structures of PPC and PPC-P. Scheme S2: Schematic diagram of how to achieve biodegradable PPC-P foams by using CO_2_; Figure S1: SEM images of cross-sections of PPC-P foams obtained at 60 °C with different saturation pressures: (a, b) 3.0 MPa, (c, d) 3.5 MPa, and (e, f) 4.0 MPa. Enlarged images in (b, d, f) show the details of the cell structure, i.e., more precise cell diameters can be gained; Figure S2: Cell size distributions of PPC-P foams obtained at 60 °C with different saturation pressure: (a) 3.0 MPa, (b) 3.5 MPa, and (c) 4.0 MPa; Figure S3: Average cell sizes, cell densities and porosities of PPC-P foams with different VER values; Figure S4: Mechanical properties of pristine PPC-P and composites; Figure S5: (a) CO_2_ uptakes of PPC-P/5%nanoCaCO_3_ composites with 3.0–4.0 MPa of saturation pressure and at 25 °C for 24 h. (b) Average cell sizes, cell densities and porosities of PPC-P/5%nanoCaCO_3_ foams with different VER obtained at 60 °C by different CO_2_ saturation pressures; Figure S6: Cell size distributions of PPC-P/5%nanoCaCO_3_ foams obtained at 60 °C with different saturation pressure: (a) 3.0 MPa, (b) 3.5 MPa, and (c) 4.0 MPa; Table S1: Statistical data of cell structure of PPC-P based foams under various foaming condition; Table S2: Compressive strength of PPC-P based foams.
