# Supplementary material for: Biodegradable and Ultra-High Expansion Ratio PPC-P Foams Achieved by Microcellular Foaming Using CO2 as Blowing Agent
Source: Nanomaterials (Basel). 2024 Jun 29;14(13):1120. doi: 10.3390/nano14131120 (PMC11243239; doi:10.3390/nano14131120)
Supplement: Supplementary file 1 [file nanomaterials-14-01120-s001.zip › nanomaterials-3070146-Supporting Information.pdf]

Supporting Information

Biodegradable and Ultra-High Expansion Ratio PPC-P Foams Achieved by Microcellular Foaming Using CO_2 as Blowing Agent

Change Wu [1], Tianwei Zhang [1], Jiaxin Liang [1], Jingyao Yin [1], Min Xiao [1], Dongmei Han [2], Sheng Huang [1], Shuanjin Wang [1,*] and Yuezhong Meng [1,2,3,4,*]

[1] The Key Laboratory of Low-carbon Chemistry & Energy Conservation of Guangdong Province/State Key Laboratory of Optoelectronic Materials and Technologies, School of Materials Science and Engineering, Sun Yat-sen University, Guangzhou 510275, China
[2] School of Chemical Engineering and Technology, Sun Yat-sen University, Guangzhou 510275, China
[3] Institute of Chemistry, Henan Academy of Sciences, Zhengzhou 450052, China
[4] Research Center of Green Catalysts, College of Chemistry, Zhengzhou University, Zhengzhou 450001, China
* Correspondence: wangshj@mail.sysu.edu.cn (S.W.); mengyzh@mail.sysu.edu.cn (Y.M.)

Fabrications and characterizations

Fabrication of PPC-P/filler composites

The dried PPC-P and 5 wt% nano-$CaCO_3$ are premixed firstly, and then the premixed materials are added into the torque rheometer (XSS-300, Shanghai Kechuang Rubber Machinery Equipment Co., LTD) for melt blending. The processing speed and melting temperature are 50 r/min and 150 °C respectively. After ~ 10 min, the PPC-P/5% nano-$CaCO_3$ composites can be obtained. Following the similar process, PPC-P/20%starch composite can be fabricated. Also, the above modified PPC-P foams are prepared using the same autoclave foaming method.

Mechanical and dynamic rheological properties

According to the standard GB/T 1040.2-2006, the tensile strength and elongation at break are obtained on a universal testing machine (CMT4504, SANS, Shenzhen, China) with a stretching rate of 5 mm/min at 25 °C and relative humidity of 50% ± 5%. The specific dimension of dumbbell specimen is 25 × 4 × 1 mm^3. At least five specimens for each sample are measured. The compression strength testing of a

rectangular with sample sizes of 20 mm×20 mm×5 mm is conducted at a crosshead speed of 5 mm/min according to GB/T 8813-2008.

The dynamic rheological properties of the pure PPC-P and modified PPC-P composites are measured by using a modular compact rheometer (MCR 302, Anton Paar, Austria) with parallel-plate geometry and 25-mm diameter plates. The measurements are operated in a shear mode at 150 °C to conduct dynamic frequency sweeps under nitrogen atmosphere. The tested specimens are circular with a dimension of 25 mm ×1 mm.

The storage modulus (G′), loss modulus (G″) and complex viscosity (η*), as a function of the angular frequency (ω) ranged from 0.01 to 100 rad/s, can be obtained. The fixed strain is set to 1% in order to guarantee that the measurements are carried out within the linear viscoelastic range of the samples investigated.

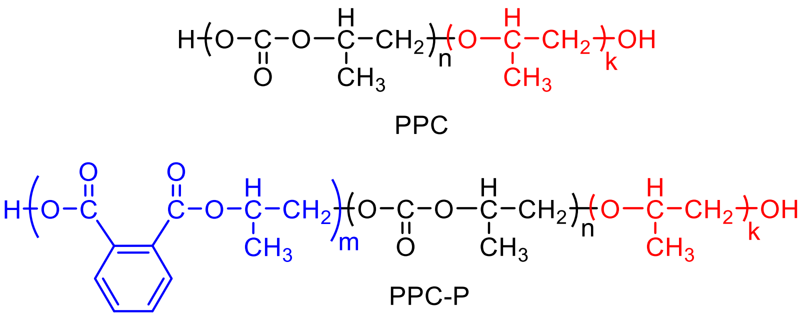

Scheme S1. Structures of PPC and PPC-P.

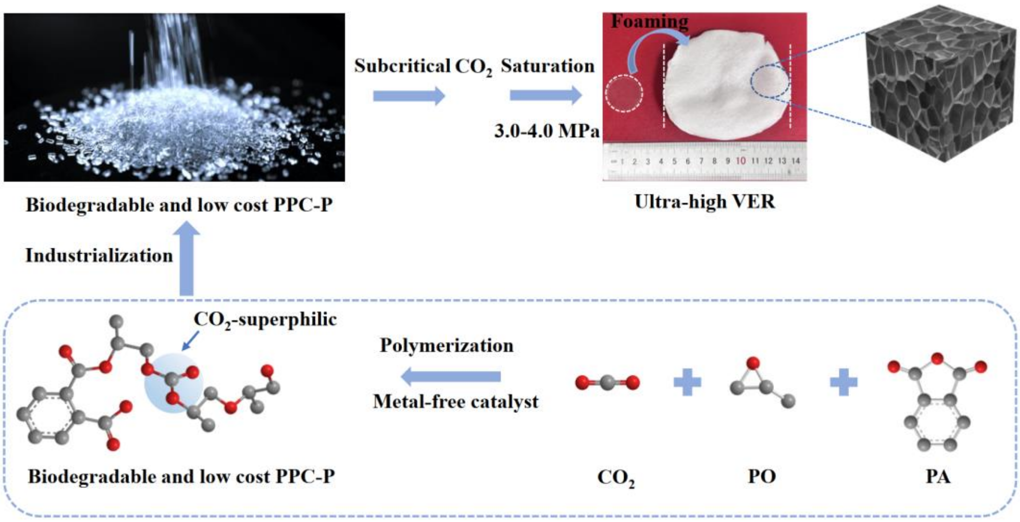

Scheme S2. Schematic diagram of how to achieve biodegradable PPC-P foams by using CO_2.

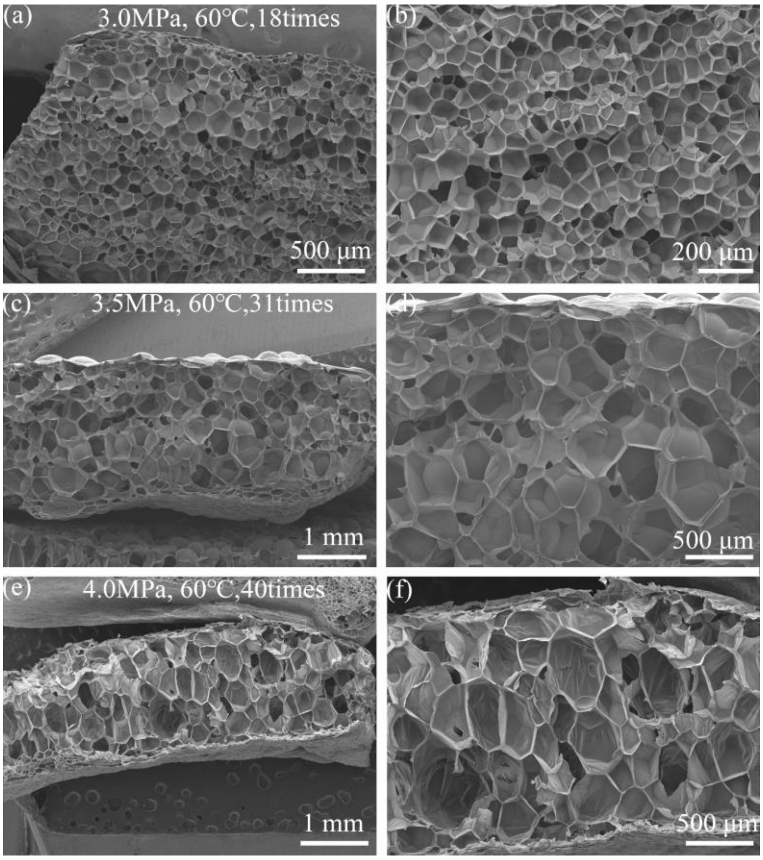

Figure S1. SEM images of cross-sections of PPC-P foams obtained at 60 °C with different saturation pressures: (a, b) 3.0 MPa, (c, d) 3.5 MPa, and (e, f) 4.0 MPa. Enlarged images in (b, d, f) show the details of the cell structure, i.e., more precise cell diameters can be gained.

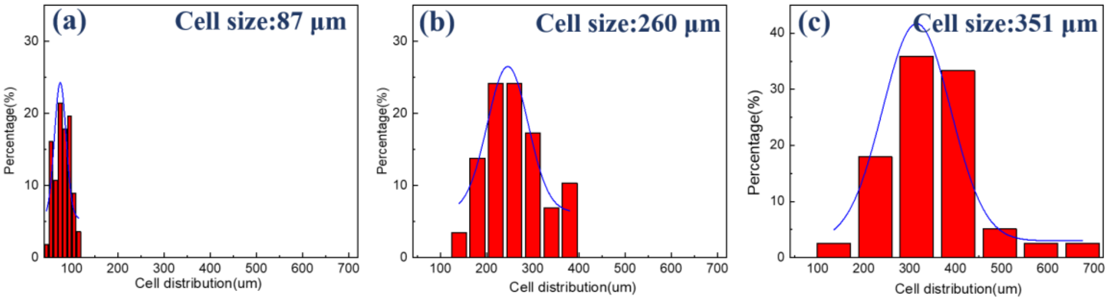

Figure S2. Cell size distributions of PPC-P foams obtained at 60°C with different saturation pressure: (a) 3.0 MPa, (b) 3.5 MPa, and (c) 4.0 MPa.

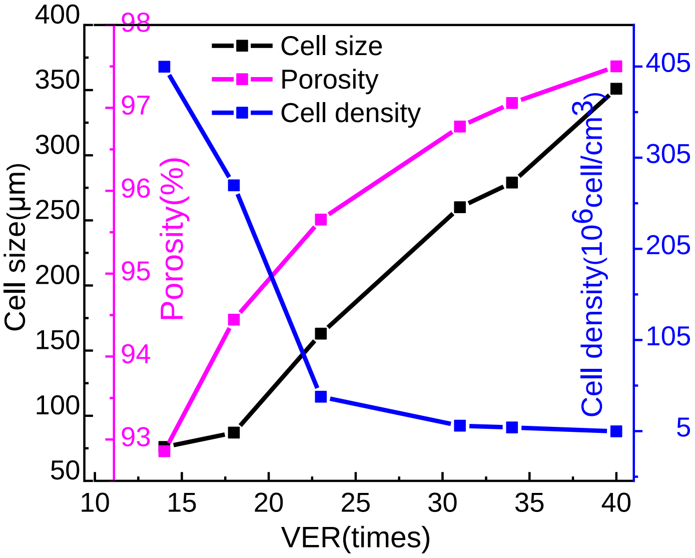

Figure S3. Average cell sizes, cell densities and porosities of PPC-P foams with different VER values.

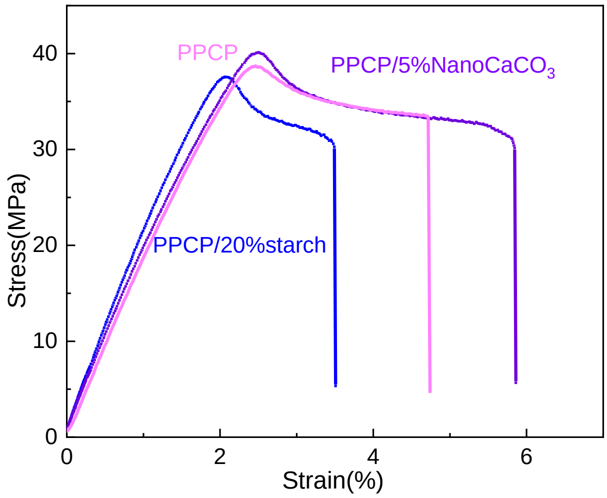

Figure S4. Mechanical properties of pristine PPC-P and composites.

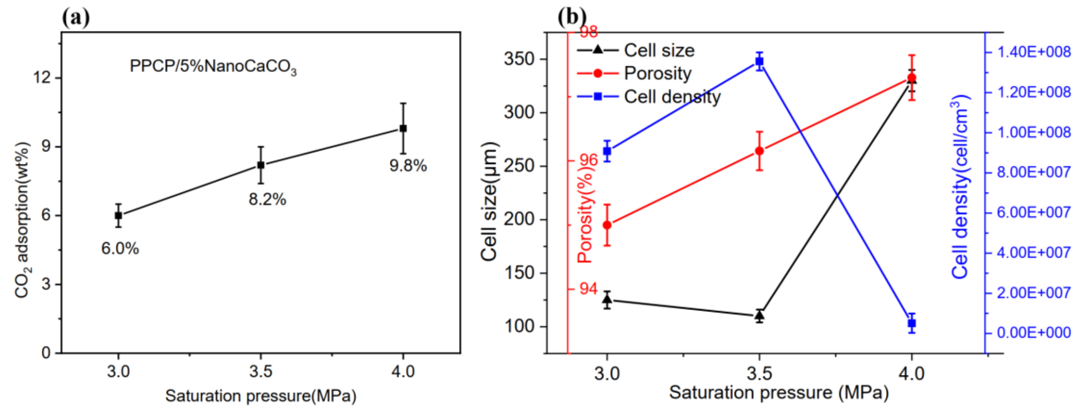

Figure S5. (a) CO_2 uptakes of PPC-P/5%nanoCaCO$_3$ composites with 3.0-4.0 MPa of saturation pressure and at 25 °C for 24 h. (b) Average cell sizes, cell densities and porosities of PPC-P/5%nanoCaCO$_3$ foams with different VER obtained at 60 °C by different CO_2 saturation pressures.

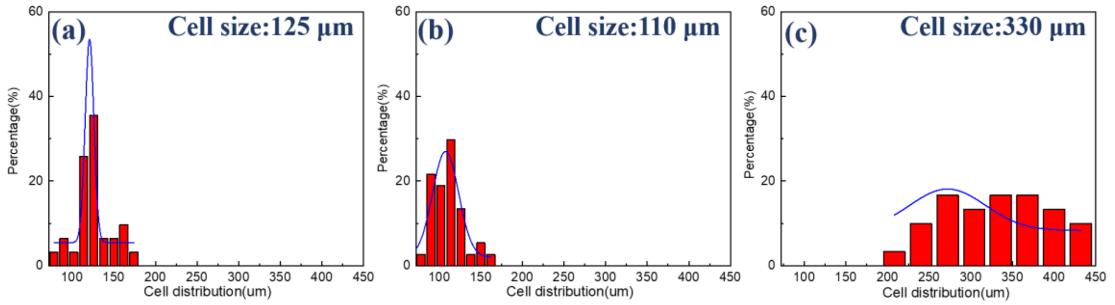

Figure S6. Cell size distributions of PPC-P/5%nanoCaCO$_3$ foams obtained at 60 °C with different saturation pressure: (a) 3.0 MPa, (b) 3.5 MPa, and (c) 4.0 MPa.

Table S1. Statistical data of cell structure of PPC-P based foams under various foaming condition.

Sample	VER (times)	Saturation pressure (MPa)	Foaming temp. (° C)	Average CO_2 adsorption (wt%)	Average cell size(μm)	Porosity (%)	Cell density (cell/cm^3)	Foam density (g/cm^3)	Nucleation density (nuclei/cm^3)
PPC-P	14	3.0	50	6.6	76.1	92.9	4.03E+08	0.089	4.51E+09
	23	3.5	50	9.7	163.4	95.7	4.19E+07	0.054	7.71E+08
	34	4.0	50	11.2	278.5	97.1	8.59E+06	0.037	2.34E+08
	18	3.0	60	6.6	87.2	94.4	2.72E+08	0.069	3.92E+09

	31	3.5	60	9.7	260.3	96.8	1.05E+07	0.040	2.60E+08
	40	4.0	60	11.2	351.3	97.5	4.30E+06	0.031	1.38E+08
PPC-P/5% nano-$CaCO_3$	20	3.0	60	6.0	124.9	95.0	9.32E+07	0.063	1.49E+09
	26	3.5	60	8.2	110.0	96.1	1.38E+08	0.048	2.87E+09
	37	4.0	60	9.8	330.4	97.3	5.15E+06	0.034	1.53E+08
PPC-P/20%Starch	13	3.0	50	6.1	124.6	92.3	9.12E+07	0.096	9.48E+08
	21	3.0	60	6.1	217.6	95.2	1.77E+07	0.060	2.97E+08

Table S2. Compressive strength of PPC-P based foams.

Samples	Compressive strength(kPa)
PPC-P/VER34	44.89
PPC-P/20%Starch/VER22	60.87
PPC-P/5%nano-$CaCO_3$/VER37	54.43
